# Nuclear receptor and VEGF pathways for gene-blood lead interactions, on bone mineral density, in Korean smokers

**DOI:** 10.1371/journal.pone.0193323

**Published:** 2018-03-08

**Authors:** Ho-Sun Lee, Taesung Park

**Affiliations:** 1 Interdisciplinary Program in Bioinformatics and Department of Statistics, Seoul National University, Gwanak 1 Gwanak-ro, Gwanak-gu, Seoul, Republic of Korea; 2 Daegu Institution, National Forensic Service, Hogukro, Waegwon-eup, Chilgok-gun, Gyeomgsamgbuk-do, Republic of Korea; Stony Brook University, Graduate Program in Public Health, UNITED STATES

## Abstract

Osteoporosis has a complex etiology and is considered a multifactorial polygenic disease, in which genetic determinants are modulated by hormonal, lifestyle, environmental, and nutritional factors. Therefore, investigating these multiple factors, and the interactions between them, might lead to a better understanding of osteoporosis pathogenesis, and possible therapeutic interventions. The objective of this study was to identify the relationship between three blood metals (Pb, Cd, and Al), in smoking and nonsmoking patients’ sera, and prevalence of osteoporosis. In particular, we focused on gene-environment interactions of metal exposure, including a dataset obtained through genome-wide association study (GWAS). Subsequently, we conducted a pathway-based analysis, using a GWAS dataset, to elucidate how metal exposure influences susceptibility to osteoporosis. In this study, we evaluated blood metal exposures for estimating the prevalence of osteoporosis in 443 participants (aged 53.24 ± 8.29), from the Republic of Korea. Those analyses revealed a negative association between lead blood levels and bone mineral density in current smokers (p trend <0.01). By further using GWAS-based pathway analysis, we found nuclear receptor (FDR<0.05) and VEGF pathways (FDR<0.05) to be significantly upregulated by blood lead burden, with regard to the prevalence of osteoporosis, in current smokers. These findings suggest that the intracellular pathways of angiogenesis and nuclear hormonal signaling can modulate interactions between lead exposure and genetic variation, with regard to susceptibility to diminished bone mineral density. Our findings may provide new leads for understanding the mechanisms underlying the development of osteoporosis, including possible interventions.

## Introduction

Osteoporosis is a skeletal disease characterized by reduced bone mass, impaired bone quality, enhanced bone resorption, and increased fracture risk. Operationally, it is defined as a bone mineral density (BMD) value ≤ -2.5 SD below the young adult mean for the population. Twin and family studies have estimated the heritability of BMD as approximately 0.85 [1]. To date, genome-wide association studies (GWAS) have identified important loci significantly correlated with disease risk/susceptibility. Recently published clinical and preclinical genetic studies of osteoporosis generally have utilized GWAS [2]. However, these findings can explain only a small portion of the heritability of osteoporosis [3], and GWAS have further shown that many phenotypes are highly polygenic, and influenced by thousands of genetic variants, each having small individual effects.

Environmental metals also significantly associate with low BMD. For example, exposure to lead (Pb) and cadmium (Cd) has been associated with a number of adverse health effects, including deficient bone mass [4–8]. Moreover, the skeletal system is thought to be highly susceptibile to these adverse effects, because the Pb body burden (roughly 75% of exposure at any given time) is retained within the mineralized compartment of bone. In addition, recent studies indicate that Cd may exert both direct and indirect actions on bone turnover, and acts directly to impair osteoblast and osteoclast function [9, 10]. Exposure to Pb and Cd can occur through occupational activities, over a lifetime, from water and food consumption and exposure to soil, dust, and air [11]. Cigarette smoking is also known as a primary source of Cd and Pb [12]. As well as active smoking, secondhand smoke also associates with increased blood Pb and Cd levels [13]. Aluminum (Al) interferes with calcium absorption, and has been reported to reduce the number of both osteroblasts and osteoclasts, resulting in less bone mineralization and bone softening (osteomalacia) [14]. However, these data have proved inconsistent, and the biological mechanisms underlying the effects of metals on bone mass, and osteoporosis, remain unclear.

Osteoporosis has a complex etiology and is considered a multifactorial, polygenic disease in which genetic determinants are modulated by environmental factors. Unexplained osteoporosis heritability could be due to gene–environment interactions, or more complex pathways involving multiple genes and other mechanisms, such as epigenetics [15]. For example, an effect of dysregulation of the *COLA1A1* gene (encoding type-1 collegen) on BMD is supported by its interaction with calcium intake [16], while the vitamin D receptor gene, *VDR* interacts with birth-weight to later modulate adult spine BMD [17]. Although recent studies suggest that circulating metals profoundly influence the adult skeleton [4, 18, 19], the interaction of specific genotypes and environmental factors, in the etiology of osteoporosis, have received limited attention. Therefore, further investigation of the interaction between environmental factors and genetic susceptibility (at the genome wide level), within the context of osteoporosis, is urgent.

The objective of this study was to identify relationships between exposure to three metals (Cd, Al, and Pb), and specific lifestyles, and the prevalence of osteoporosis. Specifically, we assessed how metal exposure and genetic susceptibility influence the onset and pathogenesis of osteoporosis, based on genome-wide association studies (GWAS). GWAS data was then subjected to gene-set-enrichment analysis (GSEA), a pathway-based analysis, to illuminate how metal exposure influences signal transduction in osteoporosis.

## Material and methods

### Study population

This study population, the Korean Association REsource (KARE) cohort, was described in detail in our previous report [20]. Briefly, a total of 8,842 participants, aged 40 to 69 years, were recruited from South Korean rural (Ansung) and urban (Ansan) communities, respectively, at baseline, from 2001 to 2002. Then, we selected 443 non-occupationally metal-exposed subjects (223 men and 221 women), aged 39 years and older, who had available blood metal level and BMD measurements. This cohort included a standardized health interview, using well-established questions, to determine the demographic and socioeconomic characteristics of all subjects. A comprehensive health examination, includuing evaluation of anthropometric indexes, such as blood pressure, and collection of biospecimens for assays, was conducted every two years, by health professionals trained by a standardized protocol. Physical activity was quantified by metabolic equivalent (MET) intensity [21]. Educational attainment was categorized into three groups: less than 7 total years (elementary school graduates), 7–9 years (middle school graduates), and more than 10 years (high school graduates). Monthly household income was also categorized into three groups: less than $1,000 USD (in 2014), $1,000–$2,000, and ≥ $2,000 [22]. This study was reviewed and approved by the Institutional Review Board of the Korean National Institute of Health.

### Measurement of BMD

BMD (g/cm^2^) and elasticity were assessed by the bone speed of sound (SOS) value [23], measured at the distal radius and midshaft tibia, using an Omnisense 7000P quantitative ultrasound (QUS) device (Sunlight Medical Ltd, Tel-Aviv, Israel). T-scores, represent comparisons of the BMD measurements of study subjects to normal values of healthy young adults of the same gender. The T-score is calculated by dividing the difference between the measured and mean SOS value in a healthy young adult population, and expressing the difference, relative to standard deviation (SD), of SOS values in a young adult population.

### Metal assessment

Blood samples were drawn into trace metal-free tubes, and analyzed by a laboratory procedure certified by the Korean Ministry of Health and Welfare. Blood lead (Pb) and cadmium (Cd) levels were determined by atomic absorption spectrometry (SpectrAA-800 Zeeman, Varian, Australia). Blood aluminum (Al) was analyzed by inductively coupled plasma optical emission spectrometry (SPECTRO Midex, SPECTRO Analytical Instruments GmbH, Germany). Commercial reference materials were used for internal quality assurance and control (Lyphochek® Whole Blood Metals Control; Bio-Rad, Hercules, CA, USA). The coefficients of variation for blood metals were ≤ 10%. External quality control was provided by the Korean Occupational Safety & Health Agency (KOSHA), and the German External Quality Assessment Scheme (G-EQUAS). The limits of detection (ng/ml) in blood for this procedure were: Pb (0.02); Cd (0.01); and Al (0.01). There were no values below limits-of-detection (LOD) levels for the three metals of interest.

### Genotyping

The KARE dataset consisted of the individual SNP chip genotypes, associated with specific epidemiological/clinical phenotypes, for studying the genetic components of Korean public health. DNA was isolated from the peripheral blood of all participants, and genotyped using the Affymetrix Genome-Wide Human SNP Array 5.0 (Affymetrix Inc, Santa Clara, CA, USA). The obtained KARE dataset passed quality control criteria and was reported in a previous GWAS publication [20]. Exclusion criteria for quality control procedures were Hardy-Weinberg equilibrium p-values < 10^−6^, genotype call rates < 95%, and minor allele frequencies (MAFs) < 0.05. All SNP chromosomal positions were updated to the human genome version 19 (hg19), as annotated in a file supplied by Affymetrix. After filtering for sample and genotype quality controls, 344,396 SNPs were available for assessing the KARE data.

### Pathway-based analysis

Pathway-based approaches, using GWAS data, are now used routinely to study complex diseases [24]. To analyze pathways interacting with blood metal levels, based on GWAS, we used an improved Gene Set Enrichment Analysis (GSEA) [25]. This approach has the advantage that genetic variant associations, mapping to any gene, provide insight into the biological functions, signal pathways, and mechanisms underlying human diseases.

In our study, 344,396 SNPs were mapped to gene-coding regions and their ± 20-kb flanking regions. Pathways consisting of <20 or >200 genes were excluded from further analysis, to reduce the multiple-testing issue, and avoid testing overly narrow or broad functional categories [25]. A false-discovery rate (FDR) was used for multiple testing correction, with q values <0.05 considered significant. An improved GSEA approach uses a comprehensive pathway/gene set database from SNP data, with pathways integrated and curated from a variety of resources, including the KEGG (Kyoto Encyclopedia of Genes and Genomes pathway database), Biocarta, and GO (gene ontology) databases [26].

### Statistical analysis

For quantifying the demographics and characteristics of subjects, data were presented as means ± standard deviations, for continuous variables, or as percentages (%), for categorical variables (Table 1). Distinctions between subjects in different groups were detected using the Kruskal-Wallis test, for non-normally distributed continuous variables (PMID: 23075015), and the chi-square test, for categorical variables.

**Table 1:** Characteristics of study participants.

Variables	N	Blood Pb (μg/L)		Blood Cd (μg/L)		Blood Al (μg/L)	
		means ± SD	P value	means ± SD	P value	means ± SD	P value^c^
Age							
≦40	23	4.05±1.86	0.51	0.76±0.97	0.13	1.36±1.01	0.42
>40, ≦60	315	4.48±1.83		1.19±1.04		1.28±1.00	
>60	105	4.38±1.68		1.05±1.25		1.16±0.68	
Sex							
Male	222	4.95±1.67	<0.01	0.97±1.00	0.01	1.19±0.85	0.85
Female in menstruation	50	3.49±1.93		1.30±1.06		1.47±1.06	
Female in menopause	121	4.09±1.80		1.30±1.25		1.20±0.86	
BMI							
<18.5	13	5.19±1.89	0.49	0.96±0.72	0.08	0.79±0.87	0.25
≥18.5, <23	124	4.40±1.88		1.34±1.37		1.33±1.06	
≥23, < 27.5	231	4.40±1.71		1.04±0.95		1.25±0.88	
≥27.5	75	4.48±1.90		1.12±1.04		1.23±0.86	
Smoking							
Never-smokers	260	4.08±1.79	<0.01	1.14±1.09	0.25	1.33±1.01	0.08
Ever-smokers^a^	174	5.00±1.69		1.11±1.13		1.15±0.80	
Current smokers	119	5.18±1.76		1.28±1.26		1.14±0.78	
Living area							
Rural	220	4.26±1.97	0.03	1.42±1.26	<0.01	1.35±0.95	0.04
Urban	223	4.61±1.60		0.86±0.81		1.17±0.91	
Education							
Elementary school or less	136	4.32±1.89	0.39	1.44±1.31	<0.01	1.26±0.90	0.69
Middle school graduate	66	4.36±1.94		1.09±0.93		1.32±0.97	
High school or higher	132	4.52±1.80		0.87±0.98		1.21±0.90	
Monthly income^b^							
<1000	183	4.41±1.88	0.63	1.32±1.16	0.01	1.24±0.88	0.34
1000–2000	144	4.54±1.81		0.94±0.91		1.34±1.03	
≥2000	104	4.42±1.68		0.94±0.94		1.16±0.86	

Values expressed as means ± SDs (standard deviations) or number (%)

^a^, formal or current

^b^10^4^ KRW, Korean Won, equivalent to $1000 USD in 2014.

^c^p value was examined by the Kruskal-Wallis test or chi square test; BMI, body mass index

For investigating possible associations between blood metal levels and BMD, we used linear regression analysis. Based on their skewed distributions, blood levels of Pb, Cd, and Al were log-transformed, for further analysis. The results of the multiple linear regression models were then expressed as regression coefficients, and standard errors adjusted for potential confounding factors. Specifically, we used the Akakie Information Criterion (AIC) for variable selection for our statistical model [27], because the AIC is a most commonly used criterion for model selection. The model with the smallest AIC value is usually chosen as a best model. Model 1 was adjusted for age, sex, and region (AIC = 1169.48). Model 2 was adjusted for age, sex, region, income, and physical activity (AIC = 1013.40). To explore the co-exposure effect of Pb, Cd, and Al on BMD, we performed multiple linear regression analyses, including two-way interaction terms such as Pb*Cd and Pb*Al, or a three-way interaction term, such as Pb*Cd*Al [28].

To test for interactions between SNPs and metals, for the purpose of identifying BMD-associated genetic variants, we tested gene-environment interactions by performing a 1-degree of freedom (1df) test of H_0_: β_int_ = 0 (i.e., the context of the linear model: *Y_i_* = β_0_+β_0_+β_1_*E_i_*+β_2_*G_i_*+β_int_G*E_*i*_+*e_i_*), where Y_i_ is the BMD T score for the individual i. The interaction term was then used to assess the significance of the interactions between genetic variants and blood Pb levels.

The critical P values for accessing the significance of interactions were calculated by Bonferroni correction (P <1.45 X 10^−7^), or false discovery rate (FDR), with a q-value ≤ 0.05 considered significant. Quantile-quantile plots of the p-values, for the joint test and interaction analysis, suggested an inflation factor of 1.16 for the interaction effect. All analyses were performed using R software version 2.11.1 (www.r-project.org). Data management, descriptive statistics for the covariates and outcome variates, and regression analyses, were conducted using the R package *Stats*.

## Results

### General characteristics of participants

Geometric mean blood heavy metal concentrations varied according to population characteristics, as shown in Table 1. The mean levels of blood Pb, Cd, and Al were 4.44 ± 1.80 μg/L, 1.14 ± 1.10 μg/L, and 1.26 ± 0.93 μg/L, respectively (median blood levels of Pb, Cd, and Al were 4.17, 0.84, and 1.1 μg/L, respectively). Blood Pb concentrations were highest in males (4.95±1.67 μg/L), followed by current smokers (5.18±1.76 μg/L), urban residents (4.61±1.60 μg/L), and high education level (4.52±1.80 μg/L). Blood Cd concentrations were also highest in current smokers (1.28±1.26 μg/L), followed by male gender (4.73 μg/L), while blood aluminum concentrations were highest in never-smokers (1.33±1.01 μg/L). Broadly, smoking status, sex, and living area associated with blood heavy metal concentration levels. Smoking was more prevalent in men, and associated with lower BMI and higher alcohol consumption (S1 Table). Smokers in women were in menstruation status. We investigated the interaction between blood metals and smoking status on BMD. However, we observed no interaction between these variables (beta = -0.64, p = 0.17 for Pb; beta = -0.09, p = 0.45 for Cd; beta = -0.16, p = 0.32 for Al).

### Correlation between blood metal levels and BMD

Correlations between blood Pb levels, alcohol consumption, and BMD T scores (as assessed by distal radius) were significant in the smoking groups (ever- and current smokers) (Table 2). BMD T score negatively associated, while alcohol consumption positively associated, with blood Pb (but not Cd or Al) levels in ever- and current smokers. Moreover, there was no significant correlation between BMD T scores and the other two metals, Cd and Al (data not shown).

**Table 2:** Correlation between blood Pb and measured parameters.

	Blood Lead (μg/L)		
factors	Never-smokers (N = 260)	Ever-smokers^a^ (N = 174)	Current smokers (N = 119)
Age, years	0.106	0.025	0.060
BMI, kg/m^2^	0.093	0.096	0.168
Physical activity (MET/hr)	-0.087	-0.0003	-0.012
Alcohol consumption (g/day)	0.058	0.267**	0.226*
BMD T score (distal radius)	-0.167*	-0.270**	-0.366***
Blood cadmium (μg/L)	-0.058	0.068	0.031
Blood aluminum (μg/L)	0.067	0.028	-0.034

Data were spearman correlation coefficients.

^a^,Smokers were former and current smokers.

BMI, body mass index; MET, metabolic equivalent; BMD, bone mineral density.

*P<0.05

**P<0.01

***P<0.001.

### Association between blood metals and BMD score

Linear regression analysis showed that levels of blood Pb and Cd, in all participants, negatively associated with BMD (Table 3), but did not correlate with lower BMD values in never-smokers, and only blood Pb negatively associated with BMD, in ever- and current smokers. For joint effects of metals, we found that co-exposure of Pb and Cd had a borderline significant association (p = 0.05) with low BMD, in current smokers, after adjustment for age, sex, region, income, and physical activity.

**Table 3:** Multivariate linear regression for blood metals (ug/L) and BMD.

	Pb		Cd		Al		Pb * Cd		Pb * Al		Pb * Cd * Al	
	(beta, SE)	P	(beta, SE)	P	(beta, SE)	P	(beta, SE)	P	(beta, SE)	P	(beta, SE)	P
All subjects												
Model 1	-1.21, 0.44	<0.01	-0.26, 0.13	0.05	-0.06, 0.18	0.74	-0.29, 0.81	0.72	-1.49, 0.93	0.11	1.87, 2.10	0.37
Model 2	-1.27, 0.48	<0.01	-0.35, 0.15	0.02	-0.15, 0.19	0.44	-0.44, 0.85	0.60	-1.51, 0.95	0.12	1.76, 2.14	0.41
Ever Smokers												
Model 1	-2.00, 0.78	0.01	-0.24, 0.19	0.21	-0.24, 0.32	0.45	-1.75, 1.43	0.22	-2.34, 2.34	0.32	3.78, 3.88	0.33
Model 2	-2.30, 0.94	0.02	-0.39, 0.23	0.09	-0.32, 0.36	0.37	-0.45, 0.85	0.60	-3.37, 2.64	0.21	2.63, 4.98	0.60
Current Smokers												
Model 1	-2.50, 0.85	<0.01	-0.13, 0.23	0.58	-0.57, 0.37	0.13	-2.54, 1.57	0.11	-1.95,2.49	0.44	2.10, 4.20	0.62
Model 2	-2.91, 1.06	<0.01	-0.34, 0.29	0.24	-0.55,0.40	0.17	-3.78, 1.90	0.05	-3.44,2.79	0.22	-1.91, 6.10	0.76
Never smoker												
Model 1	-0.78, 0.53	0.14	-0.21, 0.19	0.27	0.05, 0.21	0.80	0.75, 1.00	0.45	-1.27,1.01	0.21	0.64, 2.62	0.81
Model 2	-0.72, 0.55	0.19	-0.26, 0.19	0.18	-0.08, 0.22	0.72	0.62, 1.00	0.54	-1.35,1.00	0.18	0.33, 2.60	0.90

Model 1 is adjusted for age, sex, and regional area; model 2 is adjusted for age, sex, regional area, income, and activity.

Table 4 shows the results of linear regression models exploring the associations of the quartiles of blood Pb exposure with BMD. For ever-smokers, compared to the reference group (using the lowest interval as a reference), the quartile 3 and 4 groups of blood Pb negatively correlated with BMD (Table 4, Model 1). Using Model 2, blood Pb levels also negatively associated with BMI in ever-smokers. Current smoking more significantly associated with low BMD, after adjusting for the same models. No correlative effects of Cd or Al were found for BMD (data not shown).

**Table 4:** Linear regression of quartile of blood Pb (μg/L) and BMD.

	Quartile 1	Quartile 2	Quartile 3	Quartile 4	P trend
Ever smokers					
Model 1	reference	-0.34(-0.96~0.28)	-0.72(-1.34~-0.10)*	-0.72(-0.07~-01.38)**	<0.01
Model 2	reference	-0.45(-1.14~0.23)	-0.99(-1.71~-0.28)**	-0.86(-1.62~-0.10)*	<0.01
Current smokers					
Model 1	reference	-0.78(-0.08~-1.48)*	-1.14(-1.83~-0.45)**	-1.08(-1.86~-0.31)**	<0.01
Model 2	reference	-1.05(-1.87~0.23)*	-1.33(-2.15~-0.51)**	-1.67(-2.56~-0.78)**	<0.01

Data are expressed as beta coefficients (95% CI).

*P<0.05

**P<0.01; Model 1 is adjusted for age, sex, and regional area; model 2 is adjusted for age, sex, regional area, income, and activity.

### Interaction between blood Pb and BMD, at the pathway level

Based on the association between blood Pb levels and BMD, we further investigated interaction effects of Pb with genetic variants, with regard to BMD. With 352,228 SNPs having significant p-values for interaction terms, in the regression models, we first determined the significance of the interaction between genetic variants and blood Pb in current smokers, at the SNP DNA level. A list of SNPs associated with Pb-BMD interactions in current smokers, at *P* <5 x 10^−5^, by joint test, is provided in Table 5. Specifically, we identified SNP rs4720530, located within an intronic region of *WIPI2*, as the most significant SNP, with *P* = 3.96×10^−7^ for blood Pb-to-BMD interaction. Other interesting SNPs were located near (±2 kb) or within the loci *IGF-AS1*, *LOC107986002*, *CSRP2BP*, and *GREM1* (all nominal p values <8 x 10^−3^).

**Table 5:** Highest significant (*p*≤0.05) GWAS hits for joint and 1df interaction: Gene–blood Pb interaction on BMD in current smokers.

SNP ID	Gene	Chr	Ref/var	Position	MAF	Test of interaction	
						P_joint_	P_int_
rs4720530	Intron of *WIPI2*	7q22.1	C/T	5218800	0.358	1.97 x 10^−6^	3.96 x 10^−7^
rs1032192	Intron of *NAV2*	11p15.1	A/G	19473148	0.361	4.58 x 10^−6^	0.09
rs7103939	Intron of NAV2	11p15.1	C/T	19477837	0.361	4.58 x 10^−6^	0.09
rs10748094	Intron of *IFG-AS1*	12q15	C/T	66721583	0.249	6.31 x 10^−6^	9.96 x 10^−5^
rs7932250	Intron of *NAV2*	11p15.1	A/G	19483259	0.361	1.35 x 10^−5^	0.12
rs10207770	Intergenic near LOC107986002	2q37.3	C/T	237368042	0.056	3.13 x 10^−5^	2.54 x 10^−5^
rs1344766	Intergenic near LOC107986002	2q37.3	A/C	237368743	0.056	3.13 x 10^−5^	2.54 x 10^−5^
rs934397	Intergenic near LOC107986002	2q37.3	C/T	237371678	0.056	3.13 x 10^−5^	2.54 x 10^−5^
rs746667	Intron of *CSMD2*	1p35.1	C/G	34084766	0.051	3.334 x 10^−5^	0.27
rs13045938	Intron of *CSRP2BP*	20p11.23	A/C	18087552	0.280	3.76 x 10^−5^	2.52 x 10^−3^
rs934396	Intergenic near LOC107986002	2q37.3	A/G	237371741	0.051	3.81 x 10^−5^	5.30 x 10^−5^
rs17816285	Intergenic near *GREM1*	15q13.3	A/G	30826590	0.046	4.31 x 10^−5^	7.90 x 10^−3^
rs7161806	Intron of *NTRK3*	15q25.3	A/G	86253599	0.458	4.38 x 10^−5^	0.328
rs2702738	Intron of *NAV2*	11p15.1	C/T	129618319	0.378	4.48 x 10^−5^	0.221

However, there was no significant interaction between these SNPs and blood Pb levels (with regard to BMD), as determined by Bonferroni correction of multiple testing (nominal p-value of 1.42 x 10^−7^ of 352,228 SNPs)

Based on these findings, we tested pathway level-based interactions between blood Pb and genetic variation, according to SNPs with significant p values for enriched biological processes, in BMD. As shown in Table 6, when mapping SNPs were limited to 20kb regions flanking a gene, two pathways, nuclear receptors and the VEGF pathway, were significantly enriched, with association signals and FDRs <0.05.

**Table 6:** Pathway-based analysis of interaction between blood Pb and genetic variation, with regard to BMD, in current smokers.

Database	Pathway Name	Description	P value	FDR	Significant genes/Selected genes/All genes
Biocarta	Nuclear receptors	Nuclear receptors are transcription factors that are activated upon binding to its ligands.	<0.001	0.001	16/33/40
Biocarta	VEGF pathway	Vascular endothelial growth factor (VEGF) is upregulated by hypoxic conditions and promotes normal blood vessel formation and angiogenesis related to tumor growth or cardiac disease	<0.001	0.001	10/21/28

352,227 variants input; 185,180 variants used; 15,328 genes mapped; 230 gene sets selected

## Discussion

To our knowledge, this is the first comprehensive study of interactions between genome-wide genetic variants and blood metal levels (specifically, lead (Pb), cadmium (CD), and aluminum (Al)), with regard to susceptibility to osteoporosis. To that end, we investigated the effect of blood metal levels on BMD (bone mineral density), as an endpoint for osteoporosis). In particular, we considered the interaction between genetic variants and blood metal levels, to identify comprehensive biological pathways for susceptibility to osteoporosis, due to metal exposure. Finding no significant correlations with Cd and Al, we exclusively studied the effects of Pb on BMD.

For our study, we used data from the Korean Association REsource (KARE) cohort (PMID: 19396169), finding that the geometric mean Pb concentration of Asian subjects, 4.44 μg/L, was almost 25% of the geometric mean of 1.44 μg/dL, in U.S. adults, in the 2001–2002 National Health and Nutrition Examination Survey (NHANES) [29]. However, the U.S. Centers for Disease Control (CDC) did not include the possible influence of smoking, and we showed here that even low blood Pb levels in smokers significantly associated with reduced BMD. Although average blood Pb levels have recently declined, even chronically low Pb exposures are now well-recognized as having a persistent negative impact on human health [30]. Serious Pb-related health issues, notably in the skeleton, can occur even at blood or environmental levels below currently regulated thresholds [19]. Moreover, epidemiological studies have also reported that low levels of blood lead associate with all-cause mortality [31] and hypertension [32]. Therefore, in addition to those outcomes, chronic exposure to low Pb levels can also affect bone density, and this phenomenon requires further investigation.

Here, we show that blood Pb affects bone mineral density more strongly in smokers, in accord with many studies now showing a direct relationship between smoking and bone health. For example, in one recent study, cigarette smoking associated with low bone density, and increased the risk of hip fracture, in both women and men [33]. Others have shown a direct adverse effect of smoking on skeletal remodeling, bone cell health, and decreased BMD [2, 34]. These findings may partly relate to the influence of smoking on sex hormones, including estrogen and testosterone [35]. We postulate that this deleterious effect may be provoked by blood Pb, as smoking is a significant source of environmental Pb, demonstrating a dose-dependent relationship between tobacco and blood Pb [36]. Thus, more detailed studies of the mechanisms of synergy of smoking and circulating Pb levels, on BMD, are warranted.

Secondly, accumulating evidence shows a critical role for the Wnt/β-catenin signaling pathway in bone mass homeostasis [2]. Recent studies reported that toxic mechanisms of Pb on bone inhibit Wnt/β-catenin [37, 38] signaling, and a recent phase II trial of an antagonist of schlerostin, an inhibitor of Wnt signaling, demonstrated efficacy in increasing BMD (PMID: 25196993). However, more definitive mechanisms remain largely unknown, and very few studies have examined metal exposure as a biological mechanism of osteoporosis [37]. Researchers now recognize that gene-environmental interactions, in risk assessment for a myriad of human diseases, are crucial for studying mechanisms of disease pathogenesis [39, 40]. Toward that objective, we identified blood Pb to significantly associate with BMD in smokers, using pathway analysis for investigating interaction effects of genetic variants and blood Pb, on BMD, using a recently improved gene set enrichment analysis (GSEA). Resultantly, we found that both the nuclear receptor and vascular endothelial growth factor (VEGF) pathways associate with BMD and bone pathophysiology.

Nuclear receptors (NRs) are a family of ligand-regulated transcription factors that are activated by steroid hormones, such as estrogen and progesterone, and various lipid-soluble signaling molecules, including retinoic acid, oxysterols, and thyroid hormone [41]. NRs are a group of transcription factors that, through ligand (mostly, steroid) binding, drive adaptive gene expression responses to changes in numerous nutritional, environmental, developmental, pathophysiologic, and endocrine conditions. NRs also function as metabolic sensors that control a variety of physiological processes, including skeletal homeostasis [42]. Recently, Chen *et al*., reported a positive association between blood Pb and reproductive hormone levels, in men and postmenopausal women [43]. Moreover, experimental and epidemiological studies have shown that environmental metals (e.g., Pb) may mimic NR ligands (including hormones) as crucial regulators of development, homeostasis of male and female reproductive organs, and the maintenance of bone remodeling [44]. Growing evidence supports Pb parody of the systemic action of lipophilic hormones (e.g., thyroid, vitamin D, etc.) that play a crucial role in bone development, maintenance, and pathophysiology [45–46]. However, while other recent studies have delineated many functions of liganded NRs, including their molecular mechanisms of action on bone cells, many questions remain unaddressed.

Vascular endothelial growth factor (VEGF) is a survival factor required for effective coupling of angiogenesis and osteogenesis [47, 48]. For example, VEGF is produced by inflammatory cells, as well as mesenchymal progenitors, that are recruited to sites of bone injury [49], while also stimulating osteoclast differentiation in the bone marrow. Angiogenesis, the process mediated by VEGF, plays a key role in homeostatic responses to various toxic insults [50]. Studies similar to ours report that low Pb exposure upregulated VEGF in the bone marrow [51], increasing angiogenesis [52] and osteoclastogenesis [53]. VEGF is also upregulated in pathologies such as bone metastasis and rheumatoid arthritis [54], which also diminish BMD [55]. Recent data indicate that Pb induces VEGF synthesis via PKC/AP-1 pathway signaling, while also activating ERK signaling, both of which increase angiogenesis [56, 57] and osteoclastogenesis [58]. Therefore, further studies are required to clarify the role of Pb on VEGF biosynthesis, and other mitogenic pathways, in pathophysiology of bone metabolism.

The Wnt/β-catenin signaling pathway has emerged as a critical regulatory component of the control of bone formation and downregulation of bone resorption. It is important to understand that crosstalk between multiple signaling pathways exists to regulate bone density. For example, estrogen receptor signaling and the Wnt/β-catenin pathway act synergistically in osteogenic differentiation [59, 60]. Thus, deficiencies in these pathways contribute to low BMD. In addition, Wnt/β-catenin signaling pathway is an important mediator for tissue factor-induced VEGF production during the process of angiogenesis [61]. Therefore, we will consider how crosstalk between signaling pathways, inhibited by Pb, might downregulate BMD, in future studies.

While our findings of genetic variation and blood Pb interactions, at the levels of SNPs, and their associated pathways, were seemingly inconsistent for specific genes or chromosomal regions, it is interesting that all the interactions at these two levels implicated genes/pathways involved in angiogenesis, bone mass, and nuclear receptor signaling, suggesting that several possible mechanisms underlie the pathogenesis of osteoporosis.

One limitation of our study is the lack of replicate analyses of the interactive effects between Pb and distinct genes, on BMD. However, our results do provide biological knowledge of pathways, involving multiple genes, including responses to smoking and low-level Pb exposure, thus justifying further studies of Pb toxicity and its effects on BMD. Another limitation is the small study population. In particular, the small sample size of current smokers (n = 119) precluded stringent statistical significance. Nonetheless, we identified significant novel pathways associated with genetic variation and Pb exposure, and their effects on BMD. Further mechanistic studies are required to confirm our findings, both *in vivo* and *in vitro*. In addition, we did not consider other heavy metals that might influence BMD in current smokers. However, numerous studies indicate that Pb, Cd, and Al associate with bone mineral density, and we surmise that our studies for genetic association with metals, via the potential mechanisms we identified, can be applied to other metals.

In conclusion, even low blood Pb levels associate with the prevalence of osteoporosis, after adjusting for multiple covariates. In particular, Pb negatively associates with BMD in smokers. Using GWAS-based pathway analysis of genetic variation on BMD susceptibility, we found that the nuclear receptor and VEGF pathways (e.g., angiogenesis) were significantly enriched, with regard to blood Pb, in the prevalence of osteoporosis, in current smokers. This association evokes significant implications for the deleterious effects of environmental metals on human health, warranting further molecular and cellular experimental investigation.

## Supporting information

S1 Table Characteristics of study participants depending on smoking status. (PDF)
