# Supplementary material for: Nuclear receptor and VEGF pathways for gene-blood lead interactions, on bone mineral density, in Korean smokers
Source: PLoS One. 2018 Mar 8;13(3):e0193323. doi: 10.1371/journal.pone.0193323 (PMC5843219; doi:10.1371/journal.pone.0193323)
Supplement: S1 Table — (PDF) [file pone.0193323.s001.pdf]

Nuclear receptor and VEGF pathways for gene-blood lead interactions, on bone mineral density, in Korean smokers

Ho-Sun Lee[1,2] and Taesung Park[1*]

[1]Interdisciplinary Program in Bioinformatics and Department of Statistics, Seoul National University, Gwanak 1 Gwanak-ro, Gwanak-gu, Seoul 08826, Republic of Korea

[2]Daegu Institution, National Forensic Service, 33-14, Hogukro, Waegwon-eup, Chilgok-gun, Gyeomgsamgbuk-do, Reublic of Korea

* Corresponding author: Taesung Park, Department of Statistics, Seoul National University, Gwanak 1 Gwanak-ro, Gwanak-gu, Seoul 08826, Republic of Korea; Email: tspark@stat.snu.ac.kr; Tel. (82)2-880-8924; Fax (82)2-883-6144

Table S1. Characteristics of study participants depending on smoking status

Variable	Overall (N=443)		Smokers[a] (N=174)		Never-smoker (N=260)		P value[c]
	n	Mean±SE or percent	n	Mean±SE or percent	n	Mean±SE or percent	
Age(years)	443	53.24±8.29	174	53.39±8.33	260	53.28±8.31	0.90
Sex							0.42
Men	222	50	166	95	55	21	
Female	221	50	8	5	205	79	
Area							0.64
Urban	220	50	87	50	124	49	
Rural	223	50	87	50	136	51	
BMI(kg/m^2)	443	24.57±3.19	174	23.78±3.07	260	25.17±3.15	<0.01
Pack/year	405	9.14±15.86	145	25.54±16.87	260	0	<0.01
Education(years)							<0.01
Elementary school or less	170	39	42	24	124	48	
Middle school graduate	95	22	44	26	50	19	
High school or higher	173	39	86	50	85	33	
Montly income[b]							0.41
<1000	183	43	68	40	111	44	
1000-2000	144	33	55	32	88	35	
≥2000	104	24	47	28	56	21	
Physical activity(MET/day)	398	42.63±27.11	159	42.53±27.87	234	42.51±26.56	0.71
Total Alcohol(g/day)	420	8.37±20.14	169	18.90±28.15	246	1.26±4.68	<0.01
BMD T scores[d]	338	0.41±1.50	126	0.74±1.33	203	0.17±1.56	<0.01
BMD Z scores[d]	338	1.04±1.41	126	1.20±1.37	203	0.92±1.44	
osteopenia	35	10.4	14	11.1	21	10.3	0.58
osteoporosis	20	5.9	19	15.1	1	0.5	0.32

Values expressed as means ± SDs (standard deviations) or number (%); [a], formal or current; [b]10^4 KRW equivalent with 1000 US dollar in 2014. [c]p value was examined by the Kruskal-Wallis test or chi square test ; [d], assessed by distal radius; BMI, body mass index; MET, metabolic equivalent ;BMD, bone mineral density
